# Subtype- and Site-Specific Innervation of Melanocytic Nevi as Revealed by PGP 9.5 and CGRP Expression

**DOI:** 10.3390/medicina61101828

**Published:** 2025-10-13

**Authors:** Bruno Minigo, Marin Ogorevc, Nela Kelam, Ante Čizmić, Sandra Zekić Tomaš, Katarina Vukojević, Sandra Kostić, Dubravka Vuković, Snježana Mardešić

**Affiliations:** 1 Department of Anatomy, Histology and Embryology, University of Split School of Medicine, Šoltanska 2a, 21000 Split, Croatia; bruno.minigo@mefst.hr (B.M.); nela.kelam@mefst.hr (N.K.); katarina.vukojevic@mefst.hr (K.V.); sandra.kostic@mefst.hr (S.K.); smardesi@mefst.hr (S.M.); 2 Department of Pathology, University Hospital Split, 21000 Split, Croatia; marin.ogorevc2@gmail.com (M.O.); sandra.zekic-tomas@mefst.hr (S.Z.T.); 3 Center for Translational Research in Biomedicine, University of Split School of Medicine, Šoltanska 2a, 21000 Split, Croatia; 4 Department of Dermatovenerology, University Hospital Split, 21000 Split, Croatia; acizmic@kbsplit.hr; 5 Mediterranean Institute for Life Sciences, University of Split, 21000 Split, Croatia

**Keywords:** melanocytic nevi, cutaneous innervation, PGP 9.5, UCHL1, CGRP

## Abstract

*Background and objectives:* Melanocytic nevi are among the most common skin lesions, yet their relationship with the peripheral nervous system has remained understudied. Given the neural crest origin of melanocytes and Schwann cells, and the neurotrophic signaling capabilities of pigment cells, this study aimed to investigate the density of nerve fibers within nevi and assess how it varies with respect to histological subtype and anatomical location. *Materials and Methods:* A total of 90 nevi were analyzed, including junctional, compound, and intradermal types, distributed across the head, trunk, and limbs. Immunofluorescence staining for the pan-neuronal marker PGP 9.5 and for CGRP were performed and nerve fiber density was quantified. Statistical evaluation using two-way ANOVA revealed that both nevus type and anatomical site significantly influenced the degree of total innervation. *Results:* Junctional nevi demonstrated the highest total nerve fiber density, significantly exceeding that of compound and intradermal nevi. Likewise, nevi located on the head exhibited a significantly greater density of PGP 9.5-positive nerve fibers compared to those on the trunk and limbs. No significant correlation was observed between nevus type and location, suggesting that both factors contribute independently to the differences in innervation. CGRP-positive innervation was uniform regardless of the histological type of nevus and anatomical location. *Conclusions:* These findings likely reflect the facts that junctional nevi reside at the dermo-epidermal junction, where nerve fibers are most abundant, while the skin of the head and neck is well known to be more richly innervated than other regions. In contrast, analysis of CGRP-positive fibers suggests that the heterogeneity detected with PGP 9.5 is primarily driven by other neuronal populations. The results support the hypothesis of a dynamic relationship between nevi and the peripheral nervous system, potentially mediated by neurotrophic factors. Understanding this interaction may provide insight into nevus biology, sensory symptoms reported in some lesions, and the evolving role of nerves in the tumor microenvironment.

## 1. Introduction

Melanocytic nevi are neoplasms of the skin that form with the proliferation of pigment-producing cells—melanocytes. Nevi can be found both in the epidermis as well as in the dermis. They are considered hamartomas which are tumor-like malformations made of mature cells that are normally found in the skin [1]. The formation of some melanocytic nevi is activated by a mutation of BRAFV600E although other mutations are often involved. For example, people may develop as few as tens to sometimes even hundreds of nevi whose proliferation was initiated by well-known oncogenic mutations in the mitogen-activated protein kinase (MAPK) pathway [2,3]. Nevi appear on the skin, or rarely on the mucosa, as small macules that usually become raised as they change over time. Acquired nevi typically become apparent in childhood, and their number tends to increase through the first thirty years, and then it decreases with age [3]. From a histological perspective, melanocytic nevi are categorized based on the depth at which nevus cell nests are found. They are classified into three subtypes: junctional nevi, where nevus cells are confined to the dermal–epidermal junction; compound nevi, which contain nests in both the dermal–epidermal junction and the dermis; and intradermal nevi, in which nevus cells are located exclusively within the dermis. Accurate classification plays a crucial role in differential diagnosis and clinical management [4].

As previously mentioned, nevi begin to form through the proliferation of melanocytes that have acquired mutations. It is still unknown exactly how, that is, in which layer of the skin nevi are formed. There are several theories, each of which has its own advantages and disadvantages. The generally accepted theory describes that nevi form from melanocytes in the epidermis, which during life migrate into the dermis and thus transform from junctional to compound to intradermal. Some scientists emphasize the dermal precursor model of origin, as well as the circulating precursor model, which still need to be examined in more detail [5]. Melanocytes originate from the neural crest, a transient embryonic structure that emerges from the dorsal aspect of the neural tube and gives rise to multiple derivatives, including cranial, cardiac, vagal, trunk, and sacral cell populations [6]. The melanocytes that form the nevi of the trunk and limbs are formed by migration from the truncal neural crest. On the other hand, nevi on the head are composed of melanocytes that migrated from the cranial neural crest during embryological development [6]. More recent studies suggest that melanogenesis is hormonally and neuropeptidergically regulated notably by melanocortins acting via MC1R linking photoprotection to environmental cues and systemic signals [7]. The skin functions as a peripheral neuro-immuno-endocrine organ that senses environmental stressors (UV, microbes, chemicals, etc.) and converts them into local and systemic responses to preserve barrier integrity and whole-body homeostasis [8]. In everyday histopathological analyses of melanocytic nevi, innervation is a largely neglected component and attention is mostly focused on melanocytes, while the neural component is mostly not even mentioned. Histopathologically, a dense network of fine nerve endings penetrating the area of the surrounding dermis of intradermal nevi can be clearly seen in all samples of intradermal nevi and some studies have demonstrated the presence of myelinated neurons in compound nevi in children [9].

PGP 9.5 is a widely used pan-neuronal marker also known as ubiquitin carboxyl-terminal hydrolase 1 (UCHL1) [10]. It is a peptide found mainly in neurons and neuro-immune-endocrine cells. It is found in large quantities in the cytoplasm of cells, but its primary function is not fully known. Initial studies suggested that it participates in ubiquitin recycling. Due to its strong expression in nerve fibers, PGP 9.5 antibodies are widely used for immunohistochemical labeling of nerve fibers in various peripheral tissues. These characteristics make it an excellent candidate for determining the innervation of nevi [11].

Calcitonin gene-related peptide (CGRP) is a 37-amino-acid neuropeptide with two isoforms (alpha and beta). CGRP-positive nerve fibers are peptidergic sensory (nociceptive) fibers [12]. In the skin, CGRP-positive fibers are commonly found around blood vessels and adnexal structures, where they contribute to vasodilation and the process of neurogenic inflammation [13]. Beyond their vascular role, these fibers influence local immune and epithelial responses, including activation of mast cells, modulation of keratinocyte function and regulation of cytokine production by immune cells [14]. The clinical reference of this system is highlighted by the development of CGRP-targeted therapies, and more recent findings suggest that CGRP released from sensory fibers also support tissue repair and regeneration [15].

Given that melanocytic nevi are thought to develop progressively from junctional to intradermal forms over time, and that melanocytes derive from different regions of the neural crest depending on the anatomical site, we hypothesized that the degree of innervation may vary among nevi according to both their histological subtype and body location. To investigate this, we conducted an analysis of nerve fiber density in melanocytic nevi of various subtypes and anatomical regions, employing immunohistochemical staining with neuronal markers PGP 9.5. Through this approach, we aimed to better characterize the interaction between nevus cells and the peripheral nervous system. By providing a comprehensive overview of innervation patterns in different types of melanocytic nevi, our findings may serve as a foundation for future studies exploring the functional significance of nerve fibers within nevi and their potential role in the biology of pigmented skin lesions.

## 2. Materials and Methods

### 2.1. Sample Collection

Formalin-fixed, paraffin-embedded (FFPE) samples of melanocytic nevi were obtained from the repository of the Pathology department at the University Hospital of Split. Nevi were selected based on histopathological reports. The selection criteria included junctional, compound, and intradermal nevi from three anatomical regions: head, trunk, and limbs. Exclusion criteria were histopathological signs of atypia or inflammation, previous surgical interventions at the lesion site, and nevi from the scalp, hands or feet, due to different criteria for malignancy compared to other anatomical locations. A total of 90 nevi were included, with 10 of each nevus histological subtype per anatomical site. Each selected paraffin block was sectioned using a microtome at a 5-micrometer thickness. and tissue slices were mounted on glass slides.

### 2.2. Hematoxylin and Eosin Staining

Histopathological evaluation confirmed the benign nature of all included lesions, and no significant tissue degradation or artifacts were observed. Atypical nevi, as well as those with significant inflammatory infiltration or regression features were excluded to ensure a uniform study population and to avoid potential confounding factors while analyzing nerve fibers.

Junctional nevi displayed nests of melanocytes confined to the dermal–epidermal junction with uniform cellular morphology. Compound nevi exhibited both junctional and dermal melanocytic components, with nevus cells extending into the superficial dermis. Intradermal nevi were composed of nevus cells located exclusively within the dermis. The epidermis overlying our analyzed nevi varied from slightly flattened to acanthotic. Inflammatory infiltrates were minimal in most samples. Importantly, no mitotic activity was detected, and there were no signs of dysplasia or malignant transformation in any of our samples.

**Figure 1:**
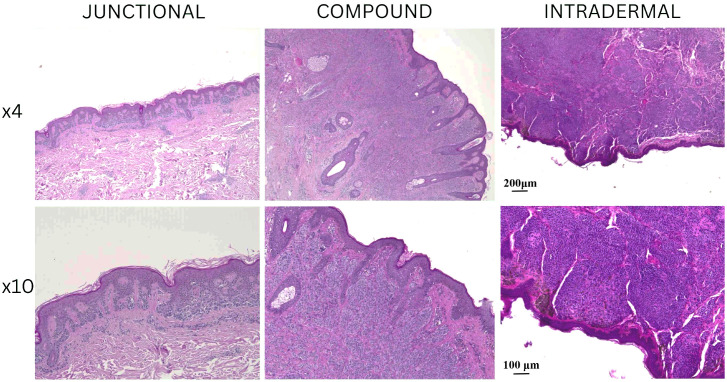
Hematoxylin and eosin (H&E) staining of melanocytic nevi. Representative examples of junctional, compound, and intradermal nevi are shown. The upper row displays images captured at 4× magnification, with a scale bar of 200 μm, illustrating the overall architecture of the lesions, while the lower row presents higher magnification views (10×), with a scale bar of 100 μm highlighting cellular morphology.

All these H&E findings confirmed the benign nature of all nevi we included in our analysis and provided a histological baseline for further immunofluorescence evaluation of nerve fiber distribution. Representative hematoxylin and eosin (H&E) stained sections of junctional, compound, and intradermal nevi are presented in Figure 1, showing the morphological characteristics at low (4×) and higher magnification (10×).

### 2.3. Immunofluorescence Staining Protocool

Nevi samples were initially subjected to deparaffinization using xylene, followed by a stepwise rehydration process through graded ethanol solutions into distilled water. Antigen retrieval was performed by heating the sections in a sodium citrate buffer (pH 6.0) for 30 min in a steam cooker. After the heating process, the slides were allowed to gradually return to room temperature. To eliminate excess reagents, the sections were rinsed thoroughly with PBS before being incubated with a protein blocking solution (Protein Block ab64226, Abcam, Cambridge, UK) for 20 min to minimize non-specific antibody binding. Following another PBS wash, an anti-PGP 9.5 rabbit monoclonal antibody (dilution 1:250, ab108986, Abcam, Cambridge, UK) was applied on one set of slides, and an anti-CGRP rabbit monoclonal antibody (1:100, ab81887, Abcam, Cambridge, UK) on a separate set of slides. Sections were incubated overnight in a humidified chamber. The next day, the slides were washed again in PBS before incubation with the corresponding secondary antibodies Alexa Flour^®^ 488 AffiniPure Anti-Rabbit (dilution 1:400, 711-545-152, Jackson Immuno Research Laboratories, Inc., West Grove, PA, USA) for one hour under the same humid conditions. After a final round of PBS washes, nuclei were counterstained using DAPI (4′,6-diamidino-2-phenylindole) for two minutes. The slides were then briefly rinsed with distilled water, air-dried for 15 min, and coverslips were mounted using Immu-Mount (Thermo Fisher Scientific, Waltham, MS, USA). To confirm staining specificity, negative control sections were prepared by omitting the primary antibodies, which resulted in no detectable fluorescence signal. The slides were examined under an Olympus BX51 fluorescence microscope (Tokyo, Japan). Microphotographs were captured using a Nikon DS-Ri2 camera (Nikon Corporation, Tokyo, Japan), and final image plate assembly was performed in Adobe Photoshop v21.0.2 (Adobe, San Jose, CA, USA).

### 2.4. Nerve Fiber Density Quantification

To evaluate the immunofluorescence signal intensity, we determined the proportion of the image occupied by the immunofluorescence signal. For each nevus sample, ten representative images were captured using a 40× objective. The image processing workflow consisted of several steps. Initially we used Adobe Photoshop (version 21.0.2, Adobe, San Jose, CA, USA) to eliminate background fluorescence by using the “levels” function. The nevus region was then manually outlined with the “Lasso” tool, extracted from the surrounding area and then placed onto a blank image of identical dimensions as the original. The processed images were imported into ImageJ (version 1.53o, NIH, Bethesda, MD, USA). We isolated the green fluorescence channel by subtracting the red channel. To enhance signal detection, duplicate images were created, and a “median” filter with radius set at 12 was applied to one of the images. The filtered images were then subtracted from the unfiltered ones, effectively isolating the positive signal. The resulting images were converted to an 8-bit format, and the fluorescence signal was segmented using the “triangle” thresholding method. The percentage of the image occupied by the fluorescent signal was determined using the “analyze particles” function. As each image contained regions with no tissue, the initially measured signal area underestimated the true fluorescence percentage. To correct for this, the total pixel count and the number of pixels corresponding to empty space were determined using the “Magic Wand” tool in Adobe Photoshop. The adjusted fluorescence area percentage was then calculated using the formula:

$$\mathrm{Corrected}\;\mathrm{area}\;\mathrm{percentage}:\;\frac{Uncorrected\;area\;percentage\;x\;total\;px}{total\;px-empty\;space\;px}$$

This value was used for our statistical analysis.

### 2.5. Statistical Analysis

All statistical analyses were performed using GraphPad Prism, version 9.5.1 (GraphPad Software, San Diego, CA, USA). Quantitative data were expressed as mean ± standard deviation (SD). Normality of data distribution was assessed using Kolmogorov–Smirnov test. To evaluate the effects of nevus subtype (junctional, compound, intradermal) and anatomical location (head, trunk, limbs) on the area percentage of PGP 9.5 positive innervation, CGRP positive innervation, the patient’s age, and nevus diameter, a two-way analysis of variance (ANOVA) was performed. The model included nevus type and location as fixed factors, and their interaction was also assessed. When main effects were found to be statistically significant, pairwise comparisons were conducted using uncorrected Fisher’s Least Significant Difference (LSD) test. Correlation between innervation density and the patient’s age or nevus diameter was determined using Pearson’s correlation coefficient. A *p*-value of <0.05 was considered statistically significant. Graphs were generated in GraphPad Prism.

## 3. Results

### 3.1. Demographic and Morphological Characteristics of the Sample

The study included a total of 90 melanocytic nevi, obtained from 42 male and 48 female patients. The mean age of participants was 44.8 years (range: 6–85 years). The average lesion diameter was 6.93 mm (range: 2–13 mm). There were no statistically significant differences between the analyzed groups of nevi regarding the patient’s sex or age, as well as nevus diameter. When analyzing the correlation between total innervation (PGP 9.5) density and clinical or demographic parameters, no significant correlation was found between innervation and lesion diameter (Pearson’s r = −0.049, *p* = 0.748), nor between innervation and patient sex. However, a weak but statistically significant positive correlation was observed between innervation and patient age (r = 0.336, *p* = 0.024), indicating a tendency toward increased innervation in older individuals. Innervation values were further compared across nevus types and anatomical locations, with significant differences identified in several subgroup analyses, detailed below. The initial morphological overview thus provided a representative and balanced dataset for subsequent comparisons.

### 3.2. Innervation Analysis

**Figure 2:**
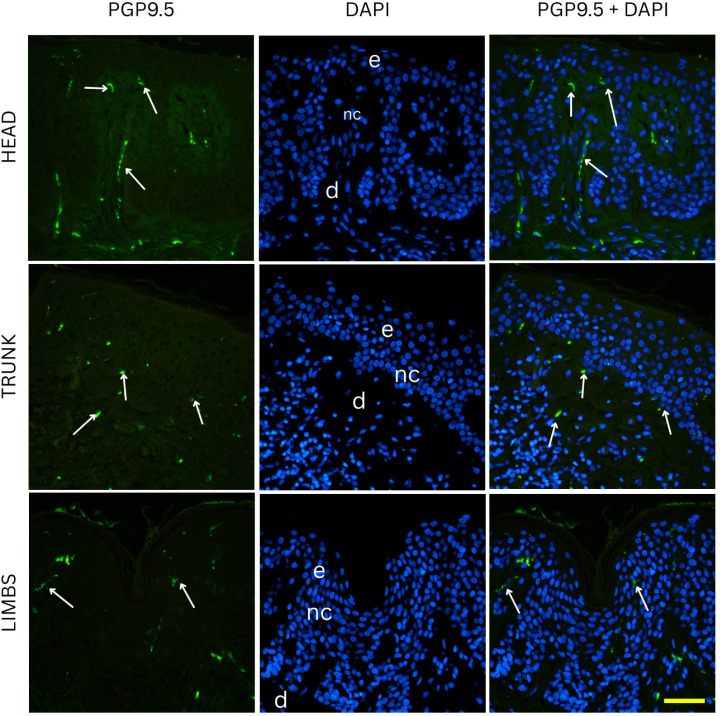
Immunofluorescence staining for PGP 9.5 and DAPI in junctional nevi from the head, trunk, and limbs. Junctional nevi show prominent innervation, particularly in samples from the head, reflecting their close proximity to the dermoepidermal junction where nerve fibers are most abundant. e—epidermis; d—dermis; nc—nevus cells; arrows—nerve fibers. Images were captured at 40× magnification, with a scale bar of 50 μm applicable to all images.

**Figure 3:**
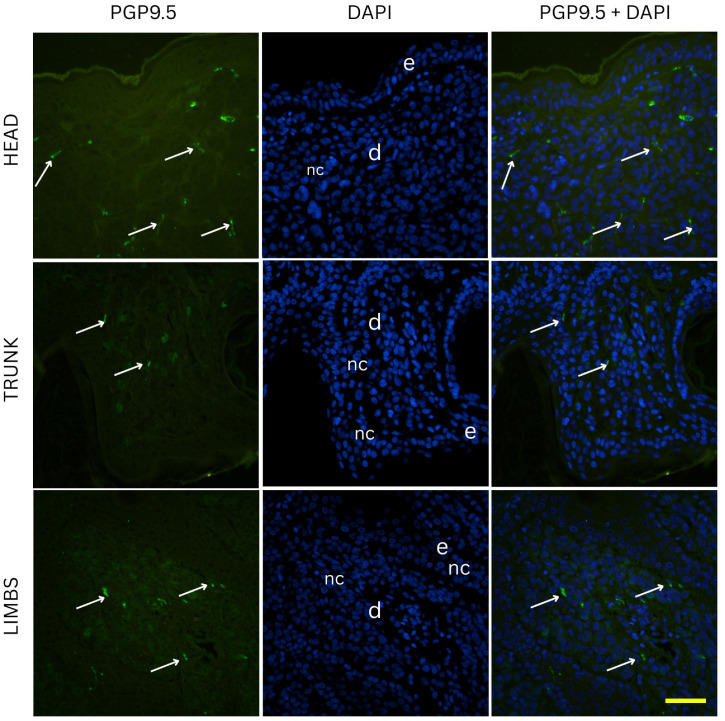
Immunofluorescence staining for PGP 9.5 and DAPI in compound nevi from the head, trunk, and limbs. Compound nevi exhibit variable nerve fiber distribution, with elements both at the dermal–epidermal junction and deeper in the dermis, reflecting their biphasic structure. e—epidermis; d—dermis; nc—nevus cells; arrows—nerve fibers. Images were captured at 40× magnification, with a scale bar of 50 μm applicable to all images.

**Figure 4:**
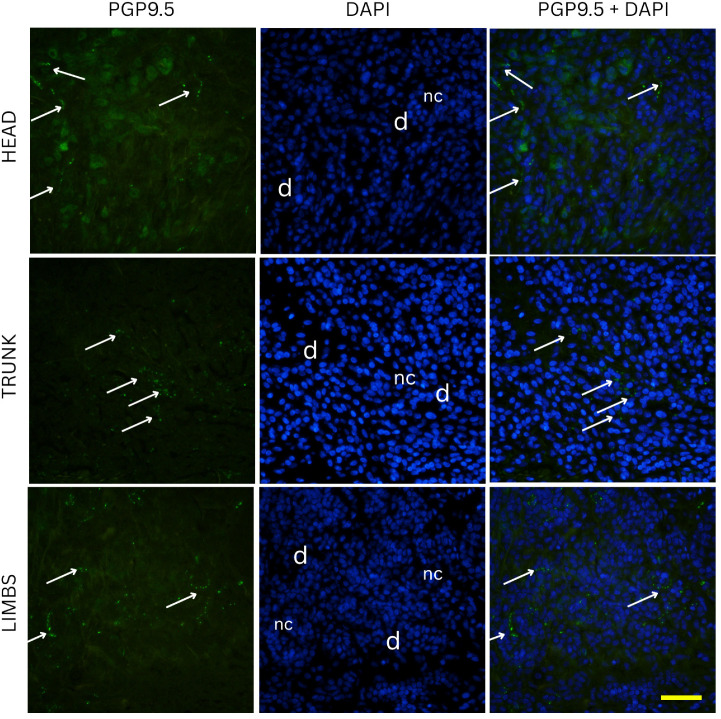
Immunofluorescence staining for PGP 9.5 and DAPI in intradermal nevi located on the head, trunk, and limbs. Images show relatively low nerve fiber density, consistent with the deeper dermal location of intradermal nevus cells, away from the superficial epidermal nerve plexus. e—epidermis; d—dermis; nc—nevus cells; arrows—nerve fibers. Images were captured at 40× magnification, with a scale bar of 50 μm applicable to all images.

Two-way ANOVA analysis of PGP 9.5-positive area percentage (as a measure of total innervation density) revealed statistically significant main effects of nevus subtype (*p* = 0.0035) and anatomical location (*p* = 0.0235), while the interaction between these two factors was not significant (*p* = 0.0641). These results indicate that both the histopathological subtype and the body region independently influence the degree of cutaneous innervation within melanocytic nevi. Representative immunofluorescence images of melanocytic nevi stained for PGP 9.5 (green) and DAPI (blue) are shown in Figure 2, Figure 3 and Figure 4, demonstrating the distribution and density of nerve fibers across different histological subtypes and anatomical locations. Figure 2 shows junctional nevi, which typically exhibit higher innervation concentrated near the dermoepidermal junction. Figure 3 includes compound (combined junctional and intradermal) nevi, displaying variable patterns of innervation that reflect their mixed architecture. Figure 4 represents intradermal nevi, characterized by their deeper dermal localization and generally lower nerve fiber density. Each figure includes representative samples from the head, trunk, and limbs to highlight both subtype- and site-specific differences in nerve fiber distribution.

**Figure 5:**
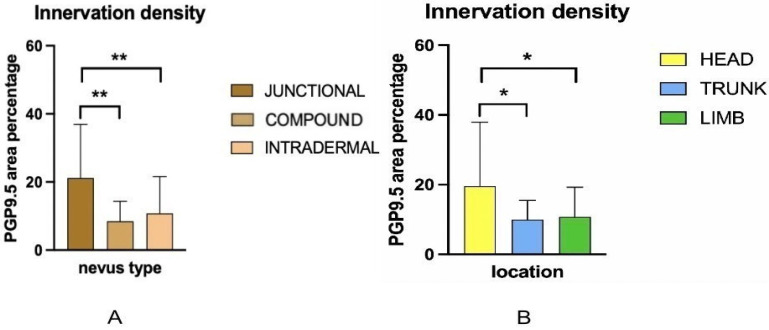
Mean innervation levels by nevus subtype (**A**) and location (**B**). * *p* < 0.05, ** *p* < 0.01.

In terms of histopathological subtype, junctional nevi had significantly greater nerve fiber density compared to both compound and intradermal nevi, with mean values approximately double that of the other two groups. Junctional nevi showed an average PGP 9.5-positive area around 20–21%, while compound and intradermal nevi ranged between 8% and 11%. These differences were statistically significant (*p* = 0.0016 for junctional vs. compound and *p* = 0.0083 for junctional vs. intradermal nevi), whereas no significant difference was observed between compound and intradermal nevi (*p* = 0.535). This pattern held across all anatomical regions. For example, on the head, junctional nevi reached an average of 35.8% ± 18.8% PGP 9.5-positive area, while intradermal nevi on the same site averaged 14.5% ± 16.1%, and compound nevi approximately 8.5% ± 6.5%. Mean innervation levels by nevus subtype are presented in Figure 5A.

When considering anatomical location, nevi located on the head exhibited significantly higher innervation compared to those on the trunk and limbs. The mean nerve fiber area percentage for head lesions was 19.63%, compared to 9.97% for the trunk and 10.78% for the limbs. Post hoc pairwise comparisons using uncorrected Fisher’s LSD revealed that the difference between head and trunk was statistically significant (*p* = 0.0133), as was the difference between head and limbs (*p* = 0.0226). However, the difference between trunk and limbs was not significant (*p* = 0.8274). These findings were consistent across nevus types and anatomical locations; junctional nevi on the head, for instance, showed the highest individual values, while compound and intradermal nevi on the trunk and limbs consistently demonstrated lower nerve densities. These quantitative differences by anatomical region are presented in Figure 5B.

Taken together, the findings demonstrate that junctional nevi, particularly when located on the head and neck, exhibit the highest levels of cutaneous innervation. Both nevus subtype and anatomical site contribute independently and significantly to the observed variations in nerve fiber density, with no statistically significant interaction detected. This suggests that the effects of nevus type and location on innervation are additive rather than interdependent.

**Figure 6:**
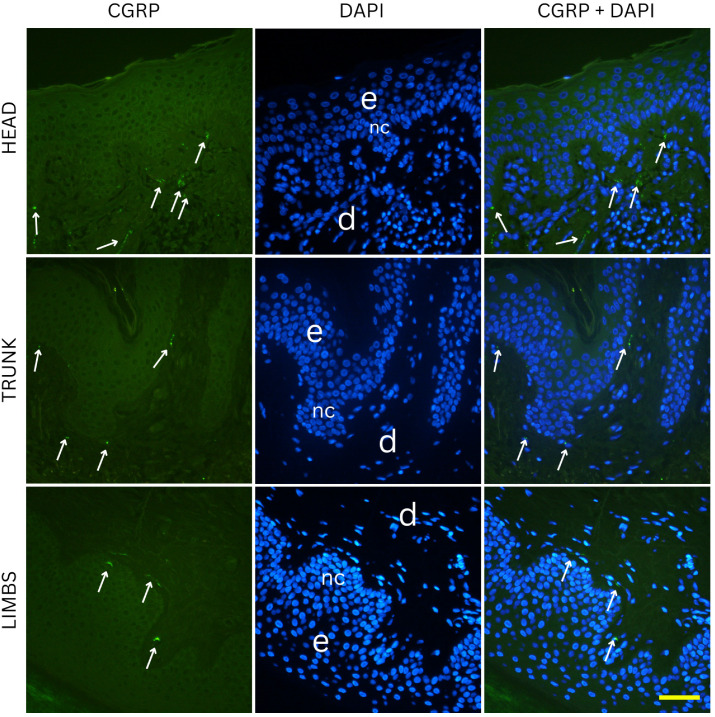
Immunofluorescence staining for CGRP and DAPI in junctional nevi from the head, trunk, and limbs. CGRP positive nerve fibers are visible along dermoepidermal junction. e—epidermis; d—dermis; nc—nevus cells; arrows—nerve fibers. Images were captured at 40× magnification, with a scale bar of 50 μm applicable to all images.

**Figure 7:**
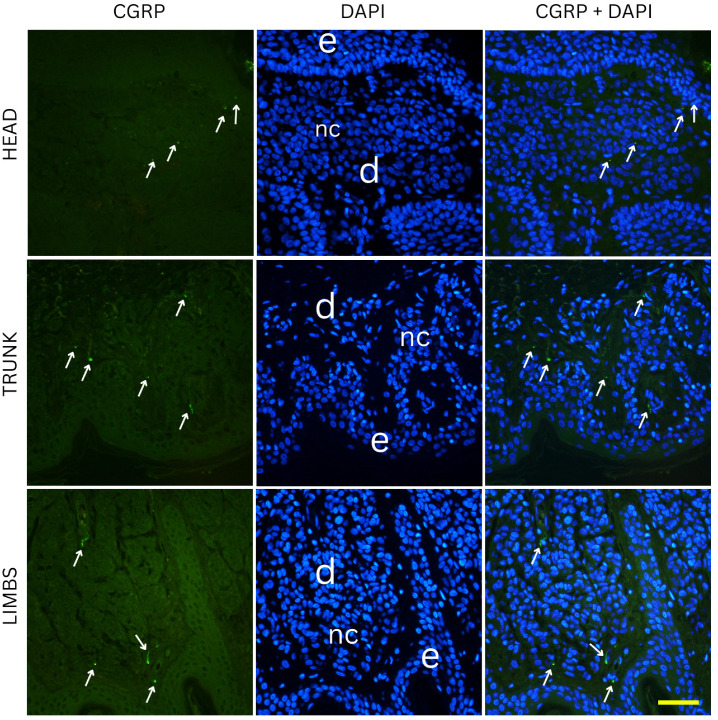
Immunofluorescence staining for CGRP and DAPI in compound nevi from the head, trunk, and limbs. Compound nevi exhibit variable nerve fiber distribution, with elements both at the dermal–epidermal junction and deeper in the dermis, reflecting their biphasic structure. e—epidermis; d—dermis; nc—nevus cells; arrows—nerve fibers. Images were captured at 40× magnification, with a scale bar of 50 μm applicable to all images.

**Figure 8:**
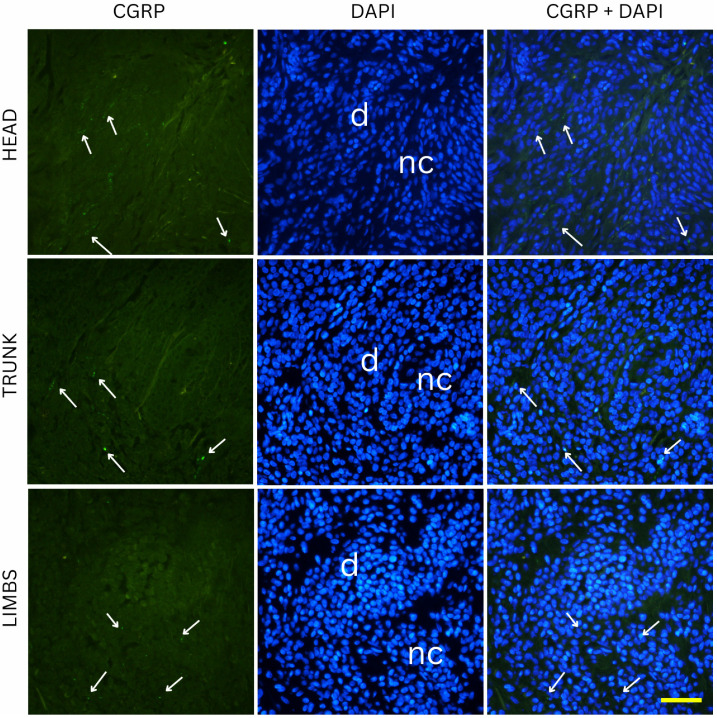
Immunofluorescence staining for CGRP and DAPI in intradermal nevi located on the head, trunk, and limbs. Images show CGRP positive nerve fibers present around melanocytes deep in the dermis. e—epidermis; d—dermis; nc—nevus cells; arrows—nerve fibers. Images were captured at 40× magnification, with a scale bar of 50 μm applicable to all images.

Two-way ANOVA analysis of CGRP immunostaining did not reveal significant differences in innervation density between various nevus types (*p* = 0.254) or anatomical locations (*p* = 0.995). Mean CGRP-positive fiber density was 6.3% in junctional nevi, 6.6% in compound nevi, and 7.3% in intradermal nevi, with no statistically significant pairwise differences (junctional vs. compound, *p* = 0.681; junctional vs. intradermal, *p* = 0.117; compound vs. intradermal, *p* = 0.234). Similarly, nevi located on the head (6.7%), trunk (6.7%) and limbs (6.8%) exhibited comparable values of CGRP-positive signal. Pairwise comparisons again showed no significant differences (head vs. trunk, *p* = 0.959; head vs. limbs, *p* = 0.964; trunk vs. limbs, *p* = 0.923). Representative immunofluorescence images for CGRP (green) and DAPI (blue) are shown in Figure 6, Figure 7 and Figure 8, illustrating the absence of evident qualitative differences in innervation patterns across intradermal, junctional and compound nevi from head, neck and trunk.

**Figure 9:**
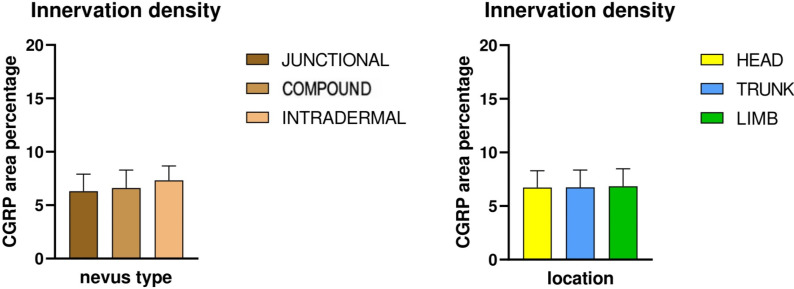
CGRP-positive innervation density expressed as mean percentage area in different nevus types (**left graph**) and anatomical locations (**right graph**).

The distribution of CGRP-positive innervation across nevus types and anatomical locations is presented in Figure 9. which shows that the density of CGRP+ fibers was similar among groups, with no statistically significant differences detected (two-way ANOVA).

Taken together, these findings indicate that the difference observed with PGP 9.5 staining are not driven by peptidergic nociceptive fibers, but are more likely attributable to other subpopulations of cutaneous nerves. This highlights a relative uniformity of CGRP-positive innervation in melanocytic nevi, regardless of histological type or body site. Such stability may imply that the functional neuro-immune roles of CGRP-positive fibers in the skin are maintained across nevi, in contrast to the broader innervation differences captured by pan-neuronal markers.

## 4. Discussion

The density of nerve fibers is highest in junctional nevi, and significantly lower in compound and intradermal nevi. This pattern can be explained by differences in both location within the skin and the natural organization of nerve fibers. Junctional nevi are positioned at the dermal–epidermal junction, close to the dense network of fine nerve fibers found in the papillary dermis. In normal skin, these fibers (highlighted by PGP 9.5 staining) either terminate at the basement membrane or enter the lower parts of the epidermis, where they contribute to sensations like pain and light touch. Because junctional nevi lie at this interface, they are exposed to a higher concentration of nerve endings.

On the other hand, intradermal nevi are located deeper in the reticular dermis, where nerve fibers are sparser and form thicker bundles. As the skin layers deepen, individual nerve endings become fewer and nerve fibers group into larger fascicles, leading to a natural decrease in innervation density [16]. Our data support this, as purely intradermal nevi (restricted to deeper dermis) showed the lowest total innervation on average, whereas nevi with a fully epidermal component (junctional) were the most innervated. Compound nevi, which have epidermal and dermal compartments, had lower innervation levels similar to that of intradermal nevi. These findings suggest that once a nevus migrates into the dermis, it loses much of the nerve richness associated with the epidermal junction region.

Biological interactions between nevus cells and nerves may help explain differences in innervation, beyond just nerve distribution in the skin. Nevus cells, derived from melanocytes, share a common origin with Schwann cells from the neural crest [17]. Because of this, melanocytes might produce neurotrophic factors that support nearby nerve fibers, similar to Schwann cells. Wrobel et al. found that benign melanocytic lesions (ordinary and dysplastic nevi) have higher nerve fiber density than normal skin. They suggested that melanocytes may promote nerve fiber survival by releasing supportive factors into their environment [17]. In other words, nevus cells might actively attract and sustain cutaneous nerve fibers via secreted growth factors. Junctional nevi might exert a stronger neurotrophic influence or simply have greater access to such factors in the epidermal milieu, whereas intradermal nevi, especially older and more differentiated ones, may have reduced production of nerve-attracting signals. It is worth mentioning that as nevi “mature” over time, they tend to migrate from the epidermal junction into the dermis, and their cellular phenotype shifts toward a more differentiated, less proliferative state [18]. Our results suggest that as nevi mature, there may be a reduction in nerve involvement. Histologically, it is known that long-standing intradermal nevi can undergo “neurotization,” where nevus cells become spindle-shaped and resemble cells of the peripheral nerve sheath [18]. However, despite this appearance, the intradermal nevi in our study showed the lowest nerve fiber density. This could imply that once nevus cells shift toward a Schwann-cell-like form, they lose the ability to strongly promote new nerve growth, or that nerve fibers naturally withdraw as the lesion ages. Overall, younger, junctional-dominant nevi remain more closely associated with nerves, while older, deeper nevi occupy a less innervated environment. Previous studies have shown that cutaneous nerves and melanocytes interact directly, forming close physical connections. Zhang et al. used microscopy to reveal synapse-like contacts between intraepidermal nerve fibers and melanocytes [7]. Functionally, sensory neuropeptides such as calcitonin gene-related peptide (CGRP) and Substance P can influence melanocyte behavior. CGRP was found to boost melanocyte proliferation and melanin production in vitro by increasing DNA synthesis and cAMP levels [7]. These findings support the idea that nerves help regulate melanocyte activity in the skin. In our study, the dense innervation seen in junctional nevi might help sustain nevus cell activity, while the reduced innervation in intradermal nevi may contribute to their more dormant, less pigmented state.

Although we cannot establish causality, our results are consistent with a dynamic relationship between nerves and pigment cells, where each influences the other [7]. We observed that nevi located on the head and neck had much higher nerve fiber densities than those on the trunk or limbs. This pattern matches what is already known about regional differences in skin nerve supply. Areas like the face and scalp are among the most heavily innervated parts of the skin. Research mapping nerve distribution has shown that the head and neck can contain nearly twice as many sensory units per square centimeter compared to the skin of the torso or legs [19]. While those results pertain to mechanoreceptors and tactile units, they reflect the broader trend that cranial skin is more densely innervated than trunk or limb skin.

The relationship between nerves and melanocytic lesions might also play a role in melanoma, the malignant transformation of nevi. Neural influences are known to affect melanoma behavior, and certain forms of melanoma show a strong tendency to grow along nerves. In particular, neurotropic melanoma, often seen in connection with desmoplastic melanoma, is marked by tumor cells spreading along nerve sheaths deep in the dermis and subcutaneous tissue. This distinct pattern of perineural invasion is a key pathological feature and, in advanced stages, can lead to nerve-related symptoms like pain or paralysis [20]. Although benign nevi do not invade nerve structures, the ability of melanoma cells to follow nerve pathways suggests that they may be guided by signals originating from nerves. In our study, we observed that melanocytic cells, especially in junctional nevi, are frequently positioned close to nerve fibers. This raises the interesting question of whether nevi with denser nerve associations could have a higher chance of progressing toward neurotropic melanoma, though there is no firm evidence supporting this idea at present. Melanoma cells have been found to produce their own nerve growth factors, which can promote their movement and support their survival. This autocrine signaling likely helps melanoma cells maintain their invasive and resilient behavior [20]. The role of nerves in tumor development is a growing focus of research. In certain settings, nerve signals may support tumor expansion and help cancers escape immune detection. Interestingly, therapies that interfere with neural pathways, such as beta-blockers, have shown potential in reducing the progression of melanoma [17,21]. In light of this, the high innervation of junctional nevi as lesions that can be precursors to melanoma is worth noting.

CGRP-positive innervation appeared remarkably uniform across nevus types and anatomical locations in our cohort, in clear contrast to the variability observed with the pan-neuronal marker PGP 9.5. Since CGRP predominantly labels peptidergic nociceptors, which arborize around dermal vessels and adnexal structures and mediate vasodilation and neurogenic inflammation, the absence of significant differences indicates that the overall changes in innervation density are unlikely to be explained by this specific fiber population [22]. The variability observed with PGP 9.5 is more likely attributable to other neuronal subsets, such as non-peptidergic sensory fibers or autonomic fibers, which are not captured by CGRP immunostaining [23]. This interpretation aligns with developmental evidence that peptidergic and non-peptidergic nociceptors are regulated by distinct neurotrophic factors, and only a subset of dorsal root ganglion neurons projecting to the skin express CGRP [24]. Across all conditions, no obvious differences in CGRP positive fibers could be observed. The preserved distribution suggests that neurovascular and neuro-immune signaling mediated by CGRP is relatively stable in benign melanocytic nevi, regardless of their depth within the skin or their anatomical location [25]. This stands in contrast to PGP 9.5-positive fibers, where subtype and site differences were evident, underscoring that the overall variation in innervation density is most likely driven by neuronal populations other than peptidergic nociceptors. From a functional perspective, this relative uniformity may indicate that CGRP-dependent pathways which include vasodilation, modulation of keratinocyte activity, and immune regulation are conserved across different nevus types [26]. Although experimental data suggest that CGRP can modulate melanocyte behavior and pigmentation under certain conditions, our findings imply that these effects are not selectively enhanced or suppressed in any benign nevus subtype [27]. Together, these results highlight that the observed heterogeneity in nevus innervation arises primarily from other neuronal subsets, and future studies incorporating additional markers will be necessary to fully characterize these differences.

This study has several limitations. First, the sample size, although large (90 nevi in total), may still have been insufficient to detect more subtle effects. A larger sample might reveal clearer regional interactions. Second, all samples were collected from a single institution (University Hospital of Split), which could limit the ability to generalize the findings to broader populations. Third, nerve density was assessed by measuring the area percentage of PGP 9.5 staining. While effective for comparing overall innervation, this method does not distinguish between sensory and autonomic fibers or confirm functional nerve connections. Future studies using different immunostainings like substance P could clarify more nerve subtypes within nevi. Fourth, we did not investigate the expression of neurotrophic factors or neuropeptides in the nevi, which could explain mechanisms underlying nerve formation. Lastly, our study focused solely on benign nevi; comparing these findings with atypical nevi or melanomas could help determine how innervation changes with malignant transformation. Despite these limitations, our results provide strong quantitative evidence that nevus innervation differs depending on subtype and anatomical site.

## 5. Conclusions

This study demonstrates that cutaneous innervation density, as assessed by PGP 9.5 immunostaining, varies significantly according to both the histological subtype and anatomical location of melanocytic nevi. Junctional nevi exhibited the highest nerve fiber density compared to compound and intradermal nevi, likely reflecting their proximity to the richly innervated dermo-epidermal junction. Anatomical location also influenced innervation, with nevi on the head showing significantly greater nerve fiber density than those on the trunk and limbs, consistent with known regional differences in skin innervation. Notably, nevus type and anatomical site contributed independently to the observed patterns, without significant interaction. In contrast, analysis of CGRP-positive fibers revealed a uniform distribution across both nevus subtypes and anatomical locations, suggesting that the heterogeneity detected with PGP 9.5 is primarily driven by neuronal populations other than CGRP. This preserved pattern of innervation implies signaling pathways mediated by CGRP remain stable in benign melanocytic nevi, regardless of their depth within the skin or anatomical site. Further research is needed to investigate nerve fiber distribution within compound nevi, specifically comparing the junctional and dermal components, as well as innervation in neurotized nevi. Comparative studies could examine innervation patterns in dysplastic or malignant lesions to assess potential roles in nevus progression or tumor microenvironment modulation. In our future studies, we aim to analyze innervation of symptomatic nevi and compare it to asymptomatic lesions that were removed due to other concerns. Additionally, correlating nerve density with mast cell infiltration, inflammation, or local neuroimmune signaling could provide insight into the interactive mechanisms between neural and immune components in skin homeostasis and neoplasia.

## Data Availability

The raw data supporting the conclusions of this article will be made available by the authors on request.

## Abbreviations

The following abbreviations are used in this manuscript:

PGP 9.5	Protein Gene Product 9.5
CGRP	Calcitonin gene-related peptide
UCHL1	Ubiquitin Carboxyl-terminal Hydrolase L1
MAPK	Mitogen-Activated Protein Kinase
FFPE	Formalin-Fixed, Paraffin-Embedded
PBS	Phosphate-Buffered Saline
DAPI	4′,6-Diamidino-2-Phenylindole
ANOVA	Analysis of Variance
SD	Standard Deviation
LSD	Least Significant Difference
NIH	National Institutes of Health
